# Genetic Analysis of Anti-Amoebae and Anti-Bacterial Activities of the Type VI Secretion System in *Vibrio cholerae*

**DOI:** 10.1371/journal.pone.0023876

**Published:** 2011-08-31

**Authors:** Jun Zheng, Brian Ho, John J. Mekalanos

**Affiliations:** Department of Microbiology and Molecular Genetics, Harvard Medical School, Boston, Massachusetts, United States of America; New England BioLabs, United States of America

## Abstract

A type VI secretion system (T6SS) was recently shown to be required for full virulence of *Vibrio cholerae* O37 serogroup strain V52. In this study, we systematically mutagenized each individual gene in T6SS locus and characterized their functions based on expression and secretion of the hemolysin co-regulated protein (Hcp), virulence towards amoebae of *Dictyostelium discoideum* and killing of *Escherichia coli* bacterial cells. We group the 17 proteins characterized in the T6SS locus into four categories: twelve (VipA, VipB, VCA0109–VCA0115, ClpV, VCA0119, and VasK) are essential for Hcp secretion and bacterial virulence, and thus likely function as structural components of the apparatus; two (VasH and VCA0122) are regulators that are required for T6SS gene expression and virulence; another two, VCA0121 and valine-glycine repeat protein G 3 (VgrG-3), are not essential for Hcp expression, secretion or bacterial virulence, and their functions are unknown; the last group is represented by VCA0118, which is not required for Hcp expression or secretion but still plays a role in both amoebae and bacterial killing and may therefore be an effector protein. We also showed that the *clpV* gene product is required for *Dictyostelium* virulence but is less important for killing *E. coli*. In addition, one *vgrG* gene (*vgrG-2*) outside of the T6SS gene cluster was required for bacterial killing but another (*vgrG-1*) was not. However, a bacterial killing defect was observed when *vgrG-1* and *vgrG-3* were both deleted. Several genes encoded in the same putative operon as *vgrG-1* and *vgrG-2* also contribute to virulence toward *Dictyostelium* but have a smaller effect on bacterial killing. Our results provide new insights into the functional requirements of *V. cholerae*'s T6SS in the context of secretion as well as killing of bacterial and eukaryotic phagocytic cells.

## Introduction

Pathogenicity of bacteria is often critically dependent on secretion systems to export toxic molecules into the environment or translocate effectors into the host cells. These secreted or injected molecules result in interference with or stimulation of host cellular processes. At least six different secretion systems (type I through type VI) have been found in Gram-negative bacterial pathogens of animals and plants [1], [2], [3]. These secretion systems are distinguished in part by the conserved structural components that define them but also by the characteristics of their substrates and the subcellular paths that their substrates take during the export process. Among these secretion systems, type VI secretion system (T6SS) has recently emerged as an exciting new topic for experimental exploration [4], [5].

T6SSs have been described in many bacterial pathogens and have been implicated in a variety of functions ranging from inter-bacterial relationships and biofilm formation, to cytotoxicity and survival in phagocytic cells [2], [6], [7], [8], [9]. T6SS gene cluster can be found in approximately 25% of all sequenced Proteobacteria and is a virulence factor in many bacteria including *Vibrio cholerae* [5], *Edwardsiella tarda* [10], *Pseudomonas aeruginosa* [4], and *Burkholderia* species [11], [12]. T6SS gene clusters contain from 15 to more than 20 genes and secrete substrates lacking N-terminal hydrophobic signal sequences [13]. Functional assays and protein localization studies suggested that the proteins encoded by T6SS gene clusters are assembled into a multi-component apparatus [10], [14], [15], [16]. Bioinformatic analysis has identified a core of 13 genes that may constitute the minimal number needed to produce a functional apparatus [17]. T6SS core components include DotU and IcmF orthologs believed to stabilize the multi-protein complex in the membrane [10], [18], and an AAA+ family ATPase called ClpV [4], [19]. ClpV associates with several other conserved T6SS proteins and its ATPase activity has been reported to be essential for T6SS function [2]. In most T6SSs, hemolysin co-regulated protein (Hcp) and valine-glycine repeat protein G (VgrG) are exported by T6SS. They are proposed to be the extracellular components of T6SS apparatus, and their secretion is co-dependent [6], [10], [20], [21], [22]. Multiple distinct T6SS gene clusters have been identified in various bacteria [4], [5], [10], [14]. In *E. tarda*, a systematic mutation of one T6SS cluster has been performed and three categories of proteins have been identified, including 12 genes required for the Hcp secretion [10].

*V. cholerae* is a Gram-negative bacterium that causes a severe, life-threatening diarrheal disease, cholera. Disease occurs when contaminated food or water is ingested, resulting in a voluminous secretory diarrhea that can lead to dehydration and death if untreated [23]. Orally ingested bacteria colonize the intestinal epithelium through expression of toxin co-regulated pili (TCP) which in turn is coordinately regulated with cholera toxin (CT), the enterotoxin responsible for the bulk of the secretory diarrhea observed during disease progression [24]. Besides these classic virulence factors, T6SS was recently implicated as a virulence determinant of *V. cholerae* using *Dictyostelium discoideum*, mouse and rabbit as model systems [5], [25], [26]. In *V. cholerae* O37 serogroup strain V52, T6SS is required for full virulence towards *Dictyostelium* amoebae and J774 macrophages [5] and for the inflammatory diarrhea in infant mice [25]. In the seventh pandemic O1 El Tor strain C6706, T6SS contributes to fecal diarrhea and intestinal inflammation in infant rabbits [26]. In addition, T6SS in *V. cholerae* displays antimicrobial properties and can cause up to a 100,000-fold reduction in *Escherichia coli* K12 survival when they co-cultivated with *V. cholerae* strain V52 on solid agar media [9].

T6SS in *V. cholerae* is responsible for the secretion of Hcp as well as three VgrG proteins (VgrG-1 to -3) [5]. VgrG-1, which functions as both a T6SS structural element and an effector protein [20], contains a C-terminal domain with strong homology to the actin cross-linking domain (ACD) of *V. cholerae* RtxA or MARTX toxin [27]. VgrG-1 causes actin cross-linking in host cells following its T6SS-dependent translocation during infection [20], [25], [28]. However, the ACD domain of VgrG-1 is not required for killing *E. coli* [9]. VgrG-1 is the only effector protein demonstrated to be translocated into eukaryotic host cells by a functional T6SS. However, recently several proteins have been identified as being likely translocated into bacterial target cells by the T6SS of *P. aeruginosa* [6].

Structural biology has revealed interesting evolutionary relationships between components of the T6SS apparatus and other dynamic structures that penetrate cell membranes [20], [21]. For example, the X-ray crystal structure of an *E. coli* VgrG-related protein predicts that VgrG proteins likely trimerize to form a complex that strongly resembles the bacteriophage T4 tail spike complex [20], [21]. The atomic structure of Hcp-related protein from *Pseudomonas aeruginosa* [4] revealed its ability to form tube-like structures in crystals that are similar to tubes formed by the N-terminal domain of trimeric VgrG proteins [21], [22]. These data suggest that in the context of a T6SS appratus, Hcp may form a tube-like structure that interacts with VgrG trimers in a complex analogous to phage tail tubes and spikes [21], [22]. Other T6SS proteins also have domains that are similar to phage tail base plate proteins [21].

In the *V. cholerae* V52 T6SS locus, VipA, VipB, VasH, VasF, ClpV, VasK, and VgrG-3 have been characterized by genetic knockout [5], [19] and bioinformatic analysis [29]. VasH is a regulator and VipA, VipB, VasF, ClpV and VasK are T6SS structural components essential for Hcp secretion [5], [19], [20]. VipA binds to VipB and they together form tubules in *E. coli* [19] that Leiman *et al.* [21] first noted have a striking resemblance to structures corresponding to the contracted tail sheath of bacteriophage T4. Thus, a phage sheath-like contraction mechanism might power the membrane penetration/secretion process of the VgrG-Hcp portion of the T6SS phage tail-like apparatus [3], [21]. ClpV ATPase activity can remodel VipA/VipB tubules *in vitro* and the ATPase activity of this protein has been reported to be a critical for substrate secretion in *P. aeruginosa* and *V. cholerae* [4], [19].

Here we report the genetic analysis of the T6SS locus of *V. cholerae* V52 in the context of its role in virulence against both *E. coli* and *Dictyostelium* amoebae. Our results show that deletion of 15 individual genes in the T6SS locus result in loss of virulence toward amoebae. Twelve of these 15 individual genes are required for secretion of Hcp but not its expression. Another two VCA0118 and VCA0122 encode a potential effector protein and a regulator, respectively. In addition, we show that several genes in the putative operons that encode *vgrG-1* and *vgrG-2* are likely involved in type VI secretion and affect T6SS-dependent virulence toward amoebae. Deletion of any of the genes predicted to encode T6SS structural elements caused significant decreases in the ability of *V. cholerae* to kill *E. coli*. Additionally, the regulatory gene *vasH*, VCA0118 and VCA0020 also contribute to T6SS-dependent killing of bacterial cells.

## Results

### Systematic mutagenesis of genes in the T6SS locus

**Figure 1:**
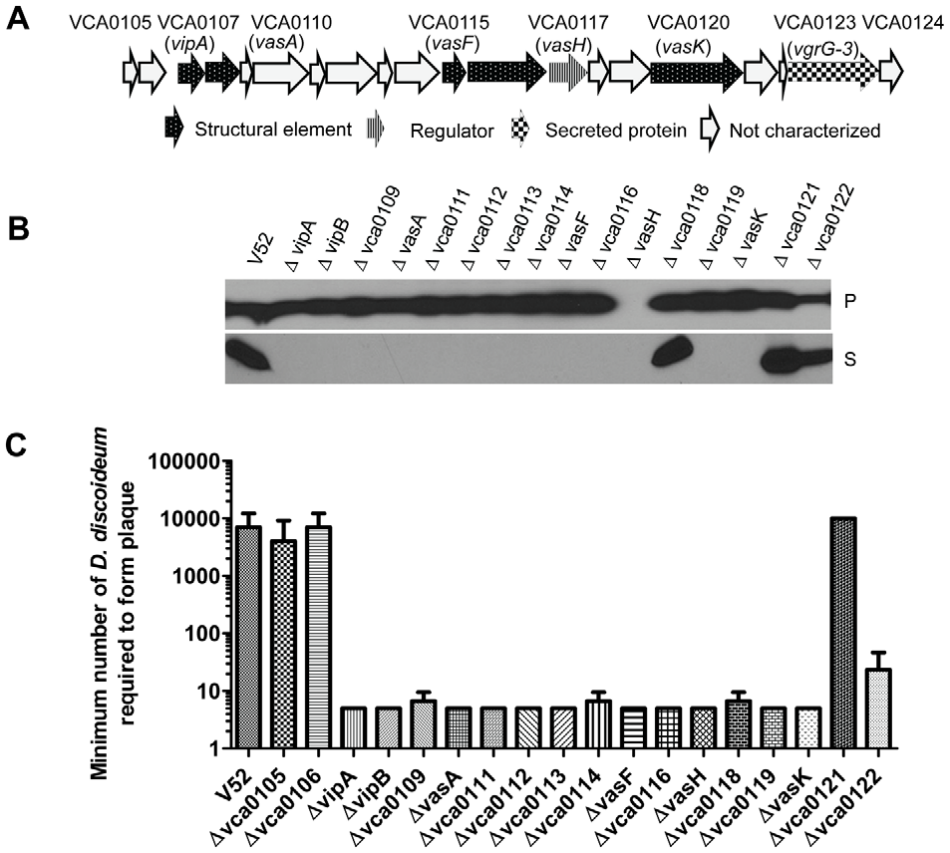
Characterization of deletion mutants in *V. cholerae* T6SS locus. (A) Schematic representation of genes organisation in *V. cholerae* T6SS locus. Genes that have been characterized were represented by arrows filled with different patterns and genes that have not been characterized were shown with empty arrows. (B) Hcp expression in bacterial pellet (P) and secretion in culture supernatant (S) in different T6SS deletion mutants were examined by western blot with anti-Hcp serum. (C) *D. discoideum* plaque formation assay for individual T6SS deletion mutant. The minimum number of *Dictyostelium* amoebae cells required for plaque formation on the lawns of *V. cholerae* wild type or mutants was shown.

The T6SS locus in *V. cholerae* contains 18 open reading frames [5]. Additionally, VCA0118, VCA0121 and VCA0122 have been found to be restricted to a smaller group of T6SS^+^ organisms and could have species-specific roles [29]. While some genetic analysis of VipA, VipB, VasF, VasH, ClpV, VasK and VgrG-3 has been performed previously [5], [19], [20], the function of other proteins encoded in this locus have not been explored in terms of their participation in both bacterial and eukaryotic T6SS-dependent killing. Thus, we began by systematically mutating all genes predicted to be in the T6SS locus of *V. cholerae* strain V52 (Fig. 1A). We generated non-polar mutations by making in-frame deletions within each gene. Thirteen mutants were successfully constructed but we were unable to delete any portion of VCA0124 for unknown reasons. VCA0124 may be essential and could encode an immunity protein for a toxic effector as has been reported for the *P. aeruginosa* T6SS [6].

In order to determine the effects of these mutations on T6SS activity, standard Hcp expression and secretion assays were performed utilizing western blot analysis and anti-Hcp antibody. Protein fractions were prepared from bacterial cell pellets and cell-free supernatant fluids from 16 T6SS mutants (Δ*vca0107 to* Δ*vca0122*). As shown in Fig. 1B, Hcp was detected in the bacterial cell pellet of all the mutants except Δ*vasH*. In addition, Hcp expression was significantly decreased in Δ*vca0122* compared to wild type and other T6SS mutants (excluding Δ*vasH*), suggesting that VCA0122 may either promote the expression of T6SS substrates as a regulator or their stabilization as a chaperone. Because Ponceau S staining of the western blot membranes showed a similar total protein level among the different mutants analyzed (data not shown), we conclude that the different levels of Hcp observed in the bacterial pellets was not due to sample loading variation during the analysis. Expression of Hcp in the cell pellet did not predict whether Hcp would be secreted by a given mutant. Thus, we examined the presence of Hcp in the supernatant fluids of mutants. As shown in Fig. 1B, Hcp could not be detected in the supernatant fluids of mutants in twelve genes (VCA0107 through VCA0116, VCA0119, and VCA0120) even though deletion of these genes did not affect Hcp expression in the cell pellet. In addition, the level of Hcp in the Δ*vca0122* mutant supernatant was significantly reduced when compared to wild type (Fig. 1B). VCA0118 and VCA0121 were not required for wild-type levels of Hcp secretion (Fig. 1B).

To further characterize the effects of these non-polar single gene mutations on *V. cholerae* virulence, we examined plaque formation by *Dictyostelium* amoebae on bacterial lawns produced with different mutant strains. We plated wild type V52 and different individual mutants on SM/5 agar plates and then 5 µl of SorC buffer containing different numbers of amoebae was deposited on each of these bacterial lawns. Plaque formation was examined after 3–5 days of incubation at 22°C, and the minimum number of *Dictyostelium* cells required for plaque formation was recorded. As shown in Fig. 1C, 10,000 *Dictyostelium* amoebae were required to form plaque on V52 wild type lawns. However, 5–10 amoebae cells were sufficient to do so on the lawns of 14 mutants carrying deletions in Δ*vca0107* through Δ*vca0120*. The minimum number of *Dictyostelium* cells required to form plaques on a Δ*vca0122* lawn was 10–50 amoebae cells, slightly higher than other mutants. In contrast, VCA0121 was not required for virulence in that it behaved identically to wild type in these amoebae plaque formation assays. The virulence of the Δ*vca0121* mutant towards *Dictyostelium* is consistent with the observation that this mutant still secretes normal levels of Hcp (Fig. 1B). Similarly, deletions of two genes (VCA0105 and VCA0106) encoded upstream of VCA0107 did not shown any significant *Dictyostelium* killing defect (Fig. 1A & C), suggesting that these genes are outside the boundary of the functional T6SS locus.

### VCA0122 controls Hcp expression at the transcriptional level

**Figure 2:**
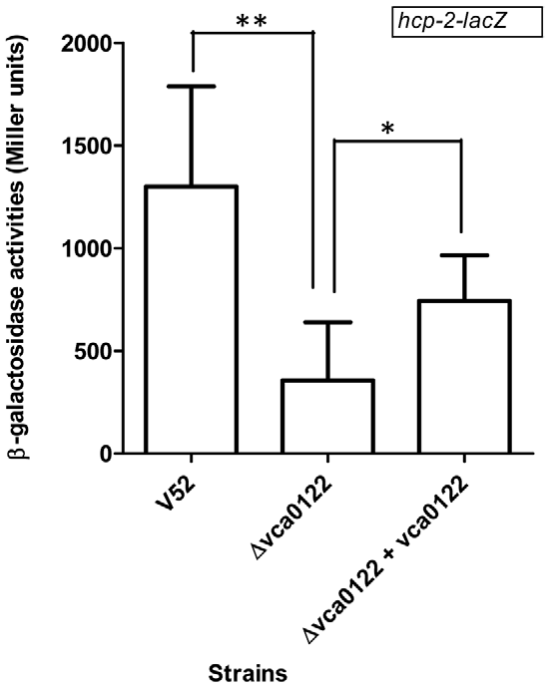
VCA0122 is a positive regulator of T6SS. The regulation of VCA0122 on T6SS substrate gene *hcp-2* was examined using *lacZ* reporter plasmid. Bacteria were cultured in LB broth. The values represent the mean ± SD. Data were analyzed with student's *t* test. ** indicates p<0.001 and * indicates p<0.05.

VCA0122 is a gene found only in *Vibrio* species and encodes a protein of 80 amino acids with no recognizable conserved domains. The Δ*vca0122* mutant expressed decreased levels of Hcp in the bacterial pellet as well as in its culture supernatant (Fig. 1B). To determine whether VCA0122 is required for transcription of T6SS genes, we examined the expression of *hcp-2* in the Δ*vca0122* mutant. We constructed on low copy-number plasmid pTL61T a transcriptional fusion between the promoter region of *hcp-2* gene and a *lacZ* reporter gene. The resulting plasmid (pTL-*hcp*) was then transformed into wild type V52 and Δ*vca0122* respectively and the β-galactosidase activities of these two strains were examined. As shown in Fig. 2, strong β-galactosidase activity was detected in the bacterial lysate of V52 with pTL-*hcp*. In contrast, this activity was decreased by about 4-fold in the Δ*vca0122* mutant. The decrease in β-galactosidase activity was partially complemented by a copy of VCA0122 provided *in trans* (Fig. 2). Taken together, these results suggest that VCA0122 is a regulator controlling the expression of the *hcp-2* locus.

### VCA0118 is not required for VgrG-1 secretion but enhances actin cross-linking activity in a macrophage cell line

**Figure 3:**
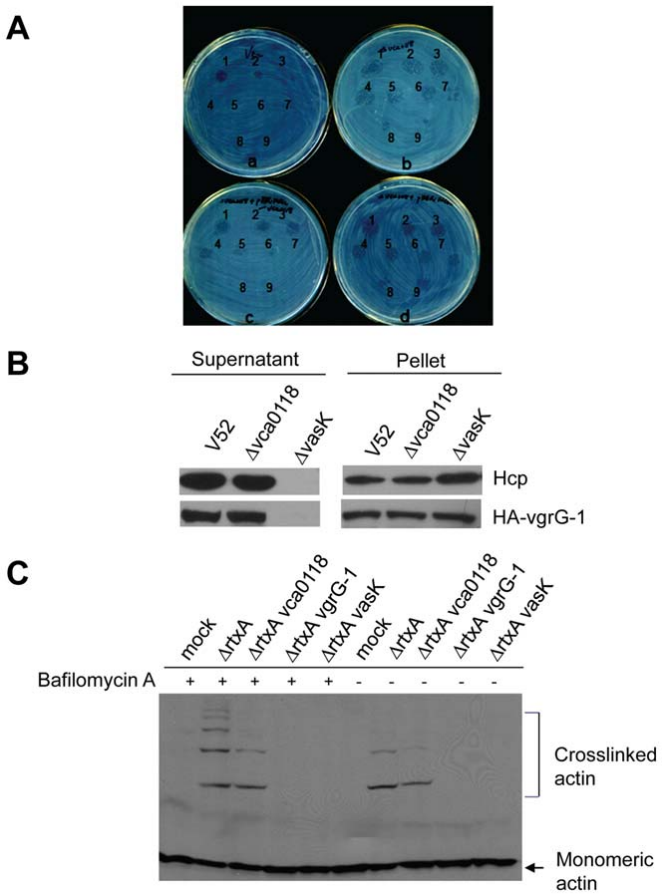
VCA0118 is a virulence factor. (A) *D. discoideum* plaque formation assay for Δ*vca0118* (plate b) and its complementation strain (plate c). The plaques formed by amoebae on the lawns of *V. cholerae* wild type (plate a) or wild type with empty plasmid (plate d) were also shown as controls. The number of *Dictyostelium* amoebae cells deposited on the plate was 1) 5×10^4^; 2) 1×10^4^; 3) 5×10^3^; 4) 1×10^3^; 5) 5×10^2^; 6) 1×10^2^; 7) 5×10^1^; 8) 1×10^1^; 9) 5×10^0^, respectively. (B) Western blot showed that VCA0118 is not required for Hcp and VgrG-1 secretion. (C) Mutation of VCA0118 reduced but does not abolish actin cross-linking by VgrG-1 with or without bafilomycin A.

VCA0118 encodes a protein that is 227 amino acids in length and contains a conserved uncharacterized domain, DUF3121. VCA0118 homologs can be found in various strains of *Vibrio* as well as many other bacterial species, including *E. coli*, *Aeromonas hydrophila*, and *Pseudomonas putida*. However, VCA0118 is absent from many other T6SS^+^ organisms [29], suggesting that it does not encode an essential structural component of the T6SS apparatus. The Δ*vca0118* mutant displayed a distinctly different phenotype than the other 15 deletion mutants in terms of its Hcp secretion profile and virulence towards *Dictyostelium* amoebae (Fig. 1B & C). Deletion of VCA0118 has no significant effect on Hcp expression or its secretion into the supernatant *in vitro* (Fig. 1B). However, Δ*vca0118* is avirulent to *Dictyostelium* amoebae. As shown in Fig. 3A, 10,000 amoebae cells are required to form plaques on wild type V52 lawns, while only 5 amoebae are needed to form a plaque on Δ*vca0118* mutant lawns. This defect was partially recovered when VCA0118 was expressed *in trans* (data not shown).

We also examined whether VCA0118 was required for the secretion of VgrG-1, the only T6SS effector required for *Dictyostelium* and macrophage virulence so far reported for *V. cholerae* [20]. As shown in Fig. 3B, Hcp and an epitope-tagged version of VgrG-1 (HA-VgrG-1) were expressed at a similar level in wild type V52, Δ*vca0118* and Δ*vasK*. Deletion of *vasK* totally abolished Hcp and VgrG-1 secretion into culture supernatant. In contrast, deletion of *vca0118* did not affect secretion of VgrG-1 and Hcp, both of which were detected in the culture supernatant at a similar level as was seen in wild type V52. Previous reports showed that V52 uses T6SS to translocate VgrG-1 into macrophage J774 cells, where the ACD domain of VgrG-1 causes actin cross-linking [20], [28]. In these studies, the addition of bafilomycin A, a specific inhibitor of vacuolar acidification, enhanced the VgrG-1-dependent, actin cross-linking activity seen in target cells [28]. Because VCA0118 is required for virulence of *V. cholerae* towards *Dictyostelium* amoebae but is not essential for the secretion of Hcp or VgrG-1, we tested if the deletion of VCA0118 affected VgrG-1-dependent actin cross-linking activity in infected J774 macrophages. For these experiments, background actin cross-linking activity was eliminated by disrupting *rtxA* in all the *V. cholerae* strains tested [20], [28]. Consistent with previous reports [20], [28], V52 Δ*rtxA* induced cross-linking of cytosolic actin in J774 cells after a 3-hour infection (Fig. 3C). Interestingly, while double Δ*rtxA vca0118* mutant still induced actin cross-linking in the presence or absence of bafilomycin A, the actin cross-linking activity we observed was less pronounced than its V52 Δ*rtxA* parental strain (Fig. 3C). As expected, no actin cross-linking was detected in macrophages infected with Δ*rtxA vasK* or Δ*rtxA vgrG-1* double mutants (Fig. 3C). These results suggest that VCA0118 contributes in some way to the delivery or activity of the actin cross-linking domain of VgrG-1 within J774 mammalian macrophages and likely within *D. discoideum* amoebae as well.

### Other genes in *vgrG-1* and *vgrG-2* operons contribute to virulence

**Figure 4:**
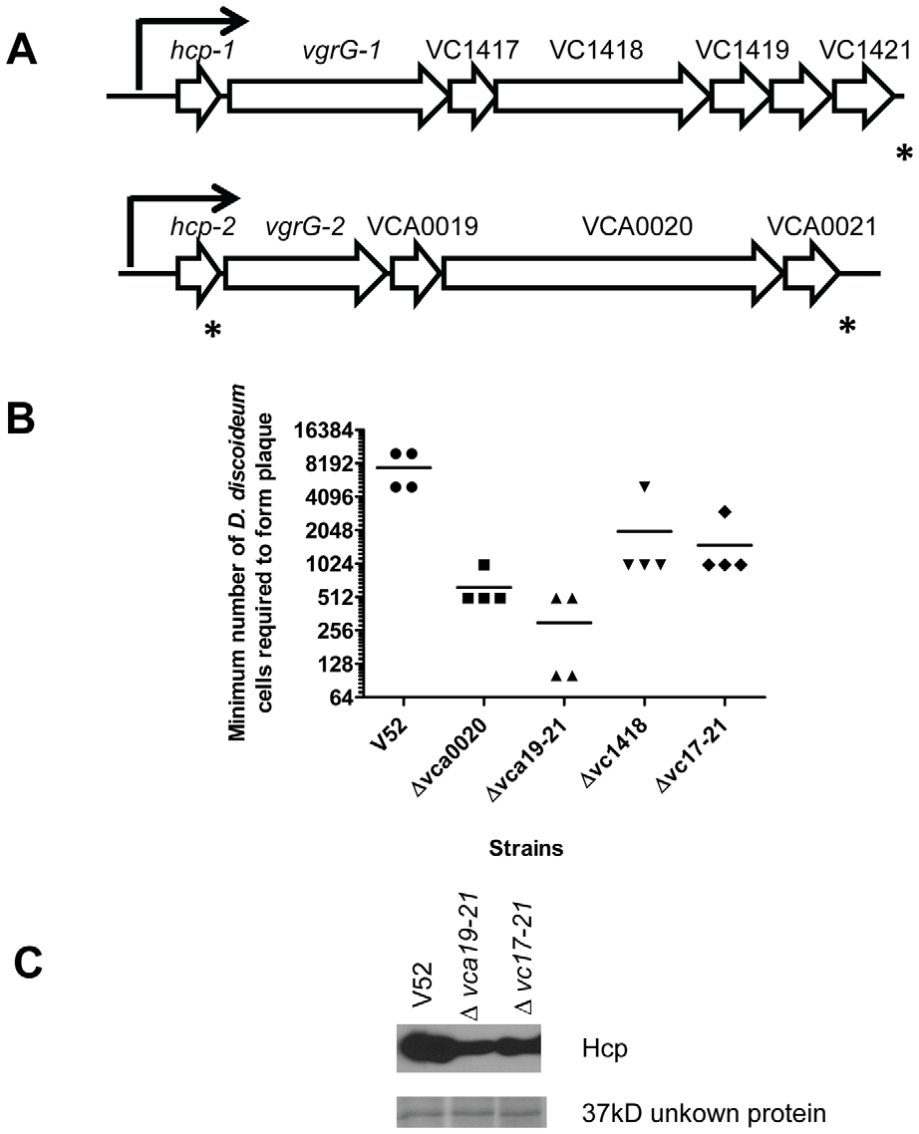
Genes encoded in the *vgrG-1* and *vgrG-2* operons contribute to *V. cholerae* virulence and Hcp secretion. (A) Schematic representation of genes organization in the putative *vgrG-1* and *vgrG-2* operons. The prompters predicted by PromScan and the terminitors predicted by TransTerm were indicated by arrows and stars respectively. (B) The minimum number of *Dictyostelieum* amoebae cells required to form plaque on the lawns of different mutants in *vgrG-1* and *vgrG-2* operons. (C) Deletion of genes in *vgrG-1* and *vgrG-2* operons affected Hcp secretion examined by western blot. A 37 kD unknown protein demonstrated by Ponceau S staining was used as a control to show the equal protein loading.

The *V. cholerae* T6SS substrates Hcp-1, VgrG-1, Hcp-2, and VgrG-2 are encoded outside of T6SS locus. Bioinformatic analysis using Promscan [30] and Transterm [31] suggest that *hcp-1*, *vgrG-1* and the five downstream open reading frames (VC1417 to VC1421) use the same promoter upstream of *hcp-1* and the same terminator downstream of VC1421 (Fig. 4A). Similarly, *vgrG-2* and the three downstream open reading frames (VCA0019 to VCA0021) are predicted to use the same promoter and terminator (Fig. 4A). All proteins encoded in these putative *vgrG-1* and *vgrG-2* operons are uncharacterized proteins and most of them do not contain conserved domains (Table S1). Interestingly, bioinformatic analysis using HHPRED [32] identified very weak structural homology of VCA0020 and VCA0021 to colicins and type III secretion system components, respectively (Table S2 and S3).

We deleted all the five genes following *vgrG-1* (VC1417 through VC1421) to get the mutant Δ*vc17–21* and deleted the single gene (VC1418) to get the mutant Δ*vc1418*. Similarly, we deleted all three genes following *vgrG-2* (VCA0019 through VCA0021) to get the mutant Δ*vca19–21* and deleted the single gene (VCA0020) to get the mutant Δ*vca0020*. We then examined the virulence of each of these mutants towards *Dictyostelium* amoebae. As shown in Fig. 4B, on average at least 7,500 amoebae cells were required for plaque formation on wild type lawns. For each of the mutants, the minimum number of *Dictyostelium* cells required to form plaques decreased significantly. The most notable of these was the Δ*vca19–21* mutant which supported plaque formation with an average of only 300 amoebae cells (Fig. 4B). These results suggest that other genes in the *vgrG-1* and *vgrG-2* putative operons might contribute to V52 virulence toward *Dictyostelium* amoebae. Since T6SS is the major *V. cholerae* virulence factor towards *Dictyostelium* and loss of T6SS activity abolished the virulence to amoebae cells [5], we tested whether the Δ*vca19–21* mutation affected T6SS function. Culture supernatant fluids from wild type V52 and the Δ*vca19–21* and Δ*vc17–21* mutants were examined for Hcp secretion by western blot. As shown in Fig. 4C, the deletion of VCA0019 through VCA0021 or VC1417 through VC1421 decreased Hcp secretion. Ponceau S staining of the western blot membrane showed a similar amount of other unknown secreted proteins had been loaded in each lane, suggesting the differences in Hcp secretion were not due to sample loading variation. A similar reduction in Hcp secretion was also observed in the single deletion mutant Δ*vca0020* (data not shown). Taken together, these results suggest that genes encoded in *vgrG-1* and *vgrG-2* putative operons also contribute to secretion by the T6SS and its function in virulence towards *Dictyostelium* amoebae.

### The antibacterial properties of different T6SS-related mutants

**Figure 5:**
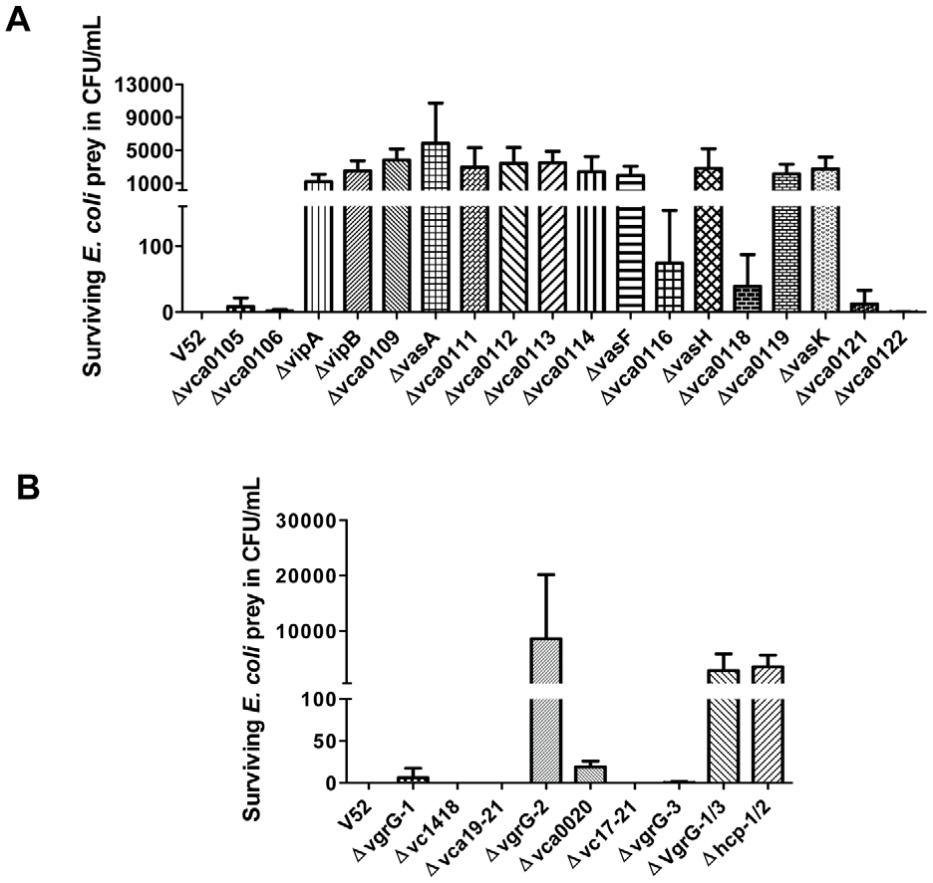
The contributions of different T6SS-related mutants on *V. cholerae* antibacterial properties. Survival of tetracycline-resistant *E. coli* CC114 was determined by measuring cfu following exposure to the streptomycin-resistant *V. cholerae* predator listed on the x-axis. The data represent three independent experiments and values given are means ± SD.

Recently, T6SS has been implicated in the killing of other bacterial species by *P. aeruginosa*, *Burkholderia thailandensis* and *V. cholerae* [6], [8], [9]. To test the contribution of each T6SS gene to the antibacterial properties of *V. cholerae*, we examined whether each of these mutants could kill *E. coli* when grown in competition on agar plates. *V. cholerae* and *E. coli* K12 were mixed, spotted onto Luria-Bertani (LB) agar plates and incubated at 37°C for 3.5 hours. Surviving *E. coli* K12 cells were then recovered from the plate and enumerated by quantitative plate counts. As previously reported [9], *V. cholerae* V52 is highly virulent toward *E. coli* K12 and literally no viable *E. coli* cells were recovered from these plates (Fig. 5A). In contrast, deletion of VCA0107 (*vipA*) through VCA0115 (*vasF*), or deletion of VCA0117 (*vasH*), VCA0119 and VCA0120 (*vasK*) resulted in complete loss of *E. coli* killing. Under the assay conditions used, T6SS-dependent killing was responsible for a 1,000 to 10,000-fold drop in the recovery of viable *E. coli* cells. Furthermore, these results were remarkably consistent with the requirement of these same genes for *Dictyostelium* amoeba killing (Fig. 1C). Thus this group of genes likely represents the set of genes required for producing a functional T6SS apparatus. Remarkably, deletion of *clpV* (VCA0116) also resulted in a clear attenuation in the bacterial killing, but not to the same degree as seen in mutants in the genes VCA0107–VCA0115, VCA0117, VCA0119 and VCA0120. Compared to these T6SS apparatus knockout mutants, approximately 30-fold fewer viable *E. coli* cells were recovered from the competitions with the Δ*clpV* mutant, indicating that significant killing activity could still be detected in the Δ*clpV* mutant. Similarly, deletion of VCA0118 caused an attenuation of *E. coli* killing activity in that about 2-fold fewer *E.coli* were recovered compared to Δ*clpV*. In contrast, loss of VCA0105, VCA0106, VCA0121 and VCA0122 had no significant effect on bacterial killing (Fig. 5A).

We then examined the antibacterial properties of the three VgrG proteins (VgrG-1 to -3). As shown in Fig. 5B, deletion of *vgrG-2* resulted in complete loss of killing. Surprisingly, we did not observed a significant bacterial killing defect in the Δ*vgrG-1* and Δ*vgrG-3* mutants. However, simultaneous deletion of both of these genes (Δ*vgrG-1/3*) produced a similar phenotype as Δ*vgrG-2* and other T6SS apparatus knockout mutants. In addition, deletion of *hcp-1* and *hcp-2* together (Δ*hcp-1/2*) also led to loss of bacterial killing. The genes in the *vgrG-1* and *vgrG-2* putative operon were also evaluated. There is no noticeable bacterial killing defect in Δ*vc1418* or Δ*vc17–21*. However, deletion of VCA0020 caused a slight attenuation in *E. coli* killing. Interestingly, this defect was not observed in Δ*vca19–21*, in which all three genes following *vgrG-2* (VCA0019 through VCA0021) were deleted (Fig. 5B).

### A Type II secretion system outer membrane protein is not required for Hcp secretion

**Figure 6:**
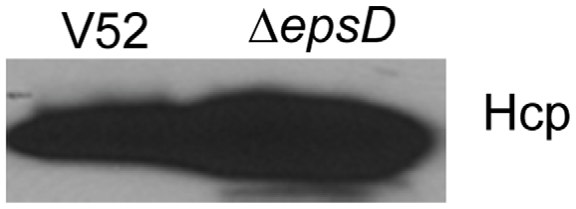
Type II secretion system outer membrane protein EpsD is not requited for Hcp secretion. Western blot of Hcp from the cell-free supernatant fluids of *V. cholerae* wild type and Δ*epsD* with anti-Hcp was shown.

A bioinformatic analysis of proteins in *V. cholerae* T6SS locus did not predict any proteins with a trans-membrane domain that might provide an outer membrane channel for secretion of T6SS effectors [29]. We thus asked whether outer membrane proteins from other secretion systems might be involved in type VI protein secretion. *V. cholerae* contains a type II secretion system in addition to its T6SS [33]. We deleted EpsD (VC2733), the outer membrane channel of type II secretion system. The secreted proteins from bacterial culture supernatant were collected and Hcp was examined by western blot. Although EpsD deficiency greatly impaired growth of *V. cholerae* (data not shown), Hcp was readily detected in the bacterial supernatant (Fig. 6), suggesting that T6SS in *V. cholerae* does not make use of type II secretion system channel for secretion of its substrates.

## Discussion

Using a *D. disoideum* model, the T6SS of *V. cholerae* was identified and designated a virulent determinant of *V. cholerae* O37 strain V52 toward amoebae [5] and toward mice [9]. More recently the T6SS of V52 was also shown to have antibacterial activity [9]. In this study, to further understand T6SS in V52, we undertook a systematic mutagenesis approach. We characterized each mutant for Hcp expression and secretion, and examined their contributions to virulence with *Dictyostelium* amoebae and *E. coli*. We identified four groups of proteins encoded in the T6SS locus: structural apparatus components, regulators, proteins of unknown function and a potential effector.

VipA, VipB, VasF, ClpV and VasK have been shown to be essential for Hcp secretion in O37 serogroup strain V52 in previous reports [5], [19]. Here we identified another 7 genes (VCA0109 through VCA0114, and VCA0119) required for Hcp secretion in V52. Deletion of these genes totally abolished Hcp secretion even though their expression levels remained similar to wild type. These mutants were also avirulent towards *Dictyostelium* amoebae and *E. coli* bacteria. Thus we speculate that collectively these genes constitute important components of the T6SS apparatus (Table 1). Deletion of T6SS structural components of the T6SS apparatus might cause defects in either secretion or translocation of T6SS effectors, either of which would likely compromise virulence.

**Table 1:** Functions of genes encoded in T6SS locus of *V. cholerae* V52.

		Required for			
Gene	Function	Hcp Secretion?	Virulence toward amoebae	Bacteria killing	References
VCA0107 (*vipA*)	Structure protein	Yes	Yes	Yes	This study and [19]
VCA0108 (*vipB*)	Structure protein	Yes	Yes	Yes	This study and [19]
VCA0109	Structure protein	Yes	Yes	Yes	This study
VCA0110 (*vasA*)	Structure protein	Yes	Yes	Yes	This study
VCA0111	Structure protein	Yes	Yes	Yes	This study
VCA0112	Structure protein	Yes	Yes	Yes	This study
VCA0113	Structure protein	Yes	Yes	Yes	This study
VCA0114	Structure protein	Yes	Yes	Yes	This study
VCA0115 (*vasF*)	Structure protein	Yes	Yes	Yes	This study and [5]
VCA0116 (*clpV*)	Structure protein	Yes	Yes	Yes/No^a^	This study and [19]
VCA0117 (*vasH*)	Regulator	Yes	Yes	Yes	This study [5]
VCA0118	Putative effector	No	Yes	Yes/No^a^	This study
VCA0119	Structure protein	Yes	Yes	Yes	This study
VCA0120 (*vasK*)	Structure protein	Yes	Yes	Yes	This study
VCA0121	Unknown	No	No	No	This study
VCA0122	Regulator	No^b^	Yes	No	This study
VCA0123 (*vgrG-3*)	Unknown/Secreted protein	No	No	Yes/No^d^	This study and [20]
VC1415 (*hcp-1* c), VCA0017 (*hcp-2* c)	Structure/Secreted protein	Yes	Yes	Yes	This study and [5]
VC1416 (*vgrG-1*)	Structure protein/Effector	Yes	Yes	Yes/No^d^	This study and [20]
VCA0018 (*vgrG-2*)	Structure/Secreted protein	Yes	Yes	Yes	This study and [20]

a *clpV* and VCA0118 mutants exhibited bacterial killing activity but at a significantly lower level than wild type.

b VCA0122 enhances Hcp expression and secretion. The VCA0122 mutant exhibited a slightly lower level of Hcp expression than wild type.

c *hcp-1* and *hcp-2* fully complement each other. Their requirement for virulence is only observed in the context of the double knock-out.

d *vgrG-1* and *vgrG-3* are able to complement each other for *E. coli* killing activity only. The double mutant is avirulent toward *E. coli*.

Interestingly, deletion of *clpV* (VCA116) has a similar effect on Hcp secretion and attenuation in *Dictyostelium* virulence as deletion of other T6SS apparatus genes but a relatively smaller effect on killing activity towards *E. coli*. These data suggest that ClpV is not essential for the process that ultimately allows *V. cholerae* to kill *E. coli* by a T6SS-dependent mechanism but rather simply increases its efficiency. Given the pore-like structure and protein translocation capability of Clp family proteins, there has been speculation that ClpV may provide the energy to translocate T6SS substrates out of bacterial cells and into target cells [4], [19]. Indeed, a ClpV ortholog has also been shown to be essential for secretion of Hcp in *P. aeruginosa* [4] and for bacterial killing [6]. ClpV of *V. cholerae* has previously been shown to be required for remodeling of the tubular complex formed by the products of VCA107 (VipA) and VCA108 (VipB) as well as efficient secretion of Hcp and VgrG-2 proteins [19]. Our results suggest that ClpV may not be required for assembly of a functional T6SS apparatus but rather simply improves the efficiency of its function. Because the tubular structure formed by VipA and VipB resembles contracted tail sheath of bacteriophage T4, Leiman *et al.* [21] first suggested that the T6SS-mediated secretion and membrane insertion process might be powered through conformational changes in VipA and VipB that mimic those occurring in phage tail contraction (rather than through the energy generated by ClpV ATP hydrolysis). It follows that, in the absence of ClpV such a phage tail-like contraction mechanism might also insert the VgrG spike and Hcp tube structure [20], [21] into closely contacted *E. coli* target cells. The non-essentiality of ClpV for V52 killing of *E. coli* is consistent with the hypothesis that ClpV is not essential for formation and function of the T6SS apparatus *per se*. Rather ClpV might play its role in the disassembly of the contracted VipA/B sheath-like structure [3], [21] (after a bacterial killing event) and thus likely improve the efficiency of killing by recycling VipA/B out of contracted sheath-like structures and back into functional non-contracted T6SS phage tail-like assemblies. A few functional T6SS assemblies might be sufficient to kill detectable numbers of *E. coli* cells in our sensitive bioassay, whereas a larger number of T6SS assemblies (or the rapid recycling of contracted sheath-like assemblies) may be needed to translocate detectable amounts of effector proteins into supernatant fluids and into amoebae or other types of target cells.

Although *V. cholerae* encodes three VgrG proteins, we found that VgrG-2 is the only VgrG protein individually essential for secretion as well as killing of amoebae and bacteria. VgrG-1 is essential for Hcp secretion and amoebae killing [5], [20], [28] but not for killing of *E. coli* (Fig. 5B) [9]. This curious fact suggests that bacterial killing may occur by a process that does not generate a large amount of extracellular Hcp and that the T6SS apparatus that mediates this killing can still be assembled in the absence of VgrG-1. It is possible that like ClpV, some T6SS-related proteins are not absolutely required for assembly or function of the apparatus, but rather alter the efficiency of its assembly or reassembly. For example, despite the fact that inactivation of VgrG-1 or VgrG-3 alone does not affect bacterial killing, inactivation of both of these genes does block killing. Because VgrG-2 interacts with VgrG-1 and VgrG-3 [20], we propose that VgrG-2 likely needs to interact with one of these other VgrG proteins to efficiently form a critical component of the T6SS apparatus such as a homo- or heterotrimer of VgrG-2.

VgrG-2 is the shortest of the three *V. cholerae* VgrG proteins and carries virtually no C-terminal extension domain. Leiman *et al.* [21] noted that short VgrG proteins are the most common form found among the hundreds of VgrG proteins encoded by microbial genomes. Thus, the short form of VgrG proteins may be the essential form of the protein conferring anti-bacterial activity through its ability to puncture cells using its needle-like β-helix C-terminal domain [21]. By analogy to phage tail-like pyocins [34], the T6SS phage tail-like spike and needle penetration would be followed quickly by lethal channel formation via insertion of the phage tube-like Hcp structure through the envelop of the target cell [21]. Recently, the T6SS of *P. aeruginosa* has been shown to have antibacterial activity through the delivery of soluble toxic effector proteins [6]. Other than the anti-eukaryotic ACD effector domain of VgrG-1 [5], [20], [25], [28], we have not uncovered a separate anti-bacterial effector in the present study and thus conclude that a functional T6SS apparatus may be sufficient for *V. cholerae* to kill *E. coli*. However, it is worth emphasizing that killing different bacterial or eukaryotic targets and using different killing mechanisms, such as membrane breaching versus delivering a separate toxic effector protein, might all require different threshold levels of T6SS assemblies. For example, pyocins kill with single hit kinetics (i.e., insertion of one pyocin can kill one bacterial cell) but soluble protein bacteriocins likely require more than one molecule to be translocated into a target cell in order to kill [34].

Four genes in the T6SS cluster of *V. cholerae* are not absolutely required for secretion of Hcp including *vasH*, VCA0118, VCA0121, and VCA0122 (Table 1). VCA0121 is also not required for virulence toward amoebae and bacteria. A bioinformatic analysis showed that both VCA0119 and VCA0121 have ImpA-rel-N superfamily domains, a feature found in inner membrane proteins participating in intracellular protein transport [29]. VCA0119 is important for T6SS-dependent secretion (Fig. 1B &C), suggesting that VCA0121 might simply be a non-functional derivative of a VCA0119 duplication. However, given their low sequence similarity (12.7% identity) it is also possible that VCA0121 was acquired by horizontal gene transfer from a non-*Vibrio* species and may have some as of yet undetermined function.

The expression of T6SS gene clusters is typically tightly regulated [1], [2], [3]. Before this study, four regulators have been shown to control T6SS in *V. cholerae*, namely, VasH, TsrA, HapR and RopN [5], [26], [35]. VasH is a sigma-54 dependent regulator encoded in T6SS locus and is required for Hcp expression [5]. TsrA is a global regulator that represses transcription of genes for T6SS and virulence factors (CT and TCP) and activates the gene for the secreted hemagglutinin protease HapA [26]. Both HapR and RpoN are positive regulators for T6SS transcription, and RpoN has been shown to directly bind to the *hcp-1*, *hcp-2*, and *vipA* promoter regions [26], [36]. Here, we found that VCA0122 is an additional regulator for *V. cholerae* T6SS. VCA0122 is an 80 amino acid protein and is not homologous to any characterized protein. Mutation of VCA0122 decreased Hcp expression at the transcriptional level presumably by affecting *hcp-2* promoter activity. Interestingly, VCA0122 plays important role in the *Dictyostelium* killing but seems to have no contribution to T6SS-dependent bacterial killing. Deletion of VCA0122 decreased but did not abolish Hcp expression and secretion (Fig. 1B). It is likely that the number of functional T6SS assemblies in Δ*vca0122* is sufficient to kill bacteria but not eukaryotic cells.

VCA0118 is a 227 amino acid-long protein and predicted to be exported [29]. It contains a DUF3121 superfamily domain, which has no characterized function. In *V. cholerae*, VCA0118 is different from all other proteins encoded in the T6SS locus in that deletion of VCA0118 did not affect the secretion of Hcp or VgrG-1, the only known eukaryotic effector protein of T6SS, but totally abolished virulence in the *Dictyostelium* amoebae model. Further investigation of actin cross-linking showed that the VCA0118 mutant was still able to cause actin cross-linking but at a lower efficiency than its V52 Δ*rtxA* parental strain (Fig. 3C). These results suggest that VCA0118 might be required for efficient translocation of VgrG-1 into host cell. Given that endocytosis of *V. cholerae* is required to observe actin cross-linking [5], [25], [26], VCA0118 may represent an extracellular adhesin that promotes binding and phagocytosis of *V. cholerae* by macrophages. VCA0118 might also participate in the translocation of T6SS effectors into target cells after phagocytosis of *V. cholerae*. If VCA0118 protein is also translocated into the host cell, then it is also possible that it might interact with VgrG-1 to promote VgrG-1 actin cross-linking activity within the host cell cytoplasm. However, considering inactivation of VCA0118 causes a total loss of virulence toward *Dictyostelium* but only slightly decreases actin cross-linking, it seems unlikely that VCA0118 functions only through its interaction with VgrG-1, as deletion of VCA0118 also decreased virulence toward *E. coli*, which does not require VgrG-1 or its ACD domain (Fig. 5B) [9], these data suggest that VCA0118 likely plays some other role besides being an enhancer of VgrG-1 toxicity. Further experiments will be needed to define the exact contribution that VCA0118 makes to the functionality of the T6SS of *V. cholerae*.

In addition to the proteins encoded in T6SS locus, we also defined several additional genes that are possibly involved in T6SS function. The genes encoded downstream of *vgrG-1* and *vgrG-2* contribute to the virulence of V52 toward *Dictyostelium*. Deletion of a single gene (VCA0020) caused attenuation of V52 virulence toward amoebae, possibly by causing a small decrease in Hcp secretion (Fig. 4C). The attenuation level in this mutant as well as a triple mutant lacking genes VCA0019–VCA0021 was relatively small compared to the decreases in virulence caused by deletion of T6SS apparatus genes, suggesting that they do not encode essential structure components of the apparatus. In the bacterial killing assay, deletion of VCA0020 showed a decreased virulence toward *E. coli*. Interestingly, this attenuation was not seen in ΔVCA19–21 in which the genes VCA0019–VCA0021 were deleted as a block. This result suggests that VCA0020 may interact with VCA0019 and VCA0021 in a complex way that affects the functionality of the T6SS apparatus in killing assays. Consistent with these results, Miyata *et al.* [37] have recently reported that mutation in VCA0020 significantly reduced virulence of *V. cholerae* toward *Dictyostelium* without altering secretion of Hcp or VgrG proteins.

In conclusion, we have shown in this study that proteins encoded in *V. cholerae* T6SS locus contribute in different ways to its functionality in Hcp secretion and the killing of amoebae and bacteria. Since T6SS is an important virulence factor in many bacterial pathogens, the characterization of gene products that are absolutely required for the functionality of the T6SS apparatus should benefit the understanding of T6SS mechanism in other bacterial species and may provide new targets for control of bacterial infectious diseases.

## Material and Methods

### Strains, plasmids and culture conditions

*V. cholerae* O37 serogroup strain V52 and its derivatives were used in this study. *E. coli* strains DH5αλ*pir*, and SM10λ*pir* were used for cloning and mating, respectively. The bacterial strains and plasmids used in this study were listed in Table 2. All bacterial strains were grown in LB broth supplemented with streptomycin (800 µg/ml), kanamycin (50 µg/ml), and carbenicillin (50 µg/ml) when necessary. Macrophage J774 cells were obtained from the American Type Culture Collection (ATCC). *D. discoideum* strain AX3 was used for bacterial virulence assay. AX3 was grown in liquid HL5 cultures or SM/5 agar plate as described by Pey [38].

**Table 2:** Strains and plasmids for this study.

Strain or plasmid	Description	Reference or source
*V. cholerae*		
V52	Wild type, Strep^R^	[5]
Δ*vca0105*	V52, in frame deletion of amino acid 1 to 61 from VCA0105	This study
Δvca0106	V52, in frame deletion of amino acid 34 to 356 from VCA0106	This study
Δ*vipA*	V52, in frame deletion of amino acid 8 to 163 from VCA0107	This study
Δ*vipB*	V52, in frame deletion of amino acid 12 to 486 from VCA0108	This study
Δ*vca0109*	V52, in frame deletion of amino acid 4 to 142 from VCA0109	This study
Δ*vasA*	V52, in frame deletion of amino acid 5 to 554 from VCA0110	This study
Δ*vca0111*	V52, in frame deletion of amino acid 14 to 313 from VCA0111	This study
Δ*vca0112*	V52, in frame deletion of amino acid 1 to 488 from VCA0112	This study
Δ*vca0113*	V52, in frame deletion of amino acid 1 to 152 from VCA0113	This study
Δ*vca0114*	V52, in frame deletion of amino acid 2 to 413 from VCA0114	This study
Δ*vasF*	V52, in frame deletion of VCA0115	[5]
Δ*clpV*	V52, in frame deletion of amino acid 3 to 850 from VCA0116	This study
Δ*vasH*	V52, in frame deletion of VCA0117	This study
Δ*vca0118*	V52, in frame deletion of amino acid 12 to 225 from VCA0118	This study
Δ*vca0119*	V52, in frame deletion of amino acid 3 to 468 from VCA0119	This study
Δ*vasK*	V52, in frame deletion of VCA0120	[5]
Δ*vca0121*	V52, in frame deletion of amino acid 1 to 421 from VCA0121	This study
Δ*vca0122*	V52, in frame deletion of amino acid 10 to 48 from VCA0122	This study
Δ*vgrG-3*	V52, in frame deletion of VCA0123	[20]
Δ*vgrG-2*	V52, in frame deletion of VCA0018	[20]
Δ*vca0020*	V52, in frame deletion of amino acid 3 to 1077 from VCA0020	This study
Δ*vca19–21*	V52, in frame deletion of genes from VCA0019 to VCA0021	This study
Δ*vgrG-1*	V52, in frame deletion of VC1416	[20]
Δ*vc1418*	V52, in frame deletion of amino acid 2 to 626 from VC1418	This study
Δ*vc17–21*	V52, in frame deletion of genes from VC1417 to VC1421	This study
Δ*hcp-1/2*	V52, in frame deletion of VCA0017 and VC1415	[5]
Δ*vgrG-1/3*	V52, in frame deletion of VCA0123 and VC1416	This study
Δ*epsD*	V52, in frame deletion of amino acid 49 to 670 from VC2733	This study
Δ*rtxA*	V52, in frame deletion of *rtxA*	This study
Δ*rtxA vca0118*	V52, in frame deletion of *rtxA* and VCA0118	This study
Δ*rtxA vgrG-1*	V52, in frame deletion of *rtxA* and *vgrG-1*	This study
Δ*rtxA vasK*	V52, in frame deletion of *rtxA* and *vasK*	This study
*E. coli*		
DH5α *pir*	*fhuA2* Δ(*argF-lacZ*)*U169 phoA glnV44 Φ80* Δ(*lacZ*)*M15 gyrA96 recA1 relA1 endA1 thi-1 hsdR17 pir*	[43]
SM10 *pir*	*thi thr leu tonA lacY supE recA-RP4-2-Tc-Mu pir*	[44]
Plasmid		
pDS132	pCVD442 modified suicide plasmid, *pir* dependent, *sac*B, Cm^R^,	[40]
pTL61T	Amp^R^, *lacZ* reporter vector	[41]
pBBR1MCS2	Km^R^, broad host range	[45]

### Non-polar deletion mutants and LacZ reporter plasmid construction

Overlap extension PCR was used to generate in-frame deletion of individual genes as described previously [39]. Briefly, for construction of a gene X deletion mutant, two PCR fragments were generated from V52 genomic DNA with the primer pair of X-for plus X-int-rev, and X-rev plus X-int-for. The resulting products generated about 1 kb fragment containing the upstream of gene X and a 1 kb fragment containing the downstream of gene X, respectively. A 20-bp overlap sequences (underlined) in the primers permitted amplification of an about 2 kb product during a second PCR with the primers X-for and X-rev. The resulting PCR product contained a deletion of the internal fragment of gene X. This PCR product was then sub-cloned into plasmid pDS132 [40] to result in a suicide plasmid construct. Non-polar deletion mutants were generated by *sacB*-based allelic exchange. The primers are listed in Table S4, and the restriction sites introduced in the primers were indicated as bold face. Mutants were verified by PCR. For construction of LacZ reporter plasmid, *hcp-2* putative promoter region was amplified from *V. cholerae* V52 genomic DNA and the PCR products were subcloned into *Xho*I and *Xba*I digested pTL61T plasmid [41].

### Protein assay

Secreted proteins were isolated from mid-log cultured *V. cholerae* (OD600 = 0.4∼0.5) in LB broth. Briefly, various overnight bacterial cultures were diluted at 1∶1,000, and were grown for about 3 to 4-hour at 37°C with shaking. The bacteria were collected by centrifuge, and the bacterial pellet was resuspended in 2× loading buffer. The bacterial culture supernatants were sterilized by passing through a 0.2 µm filter, and proteins were precipitated with trichloroacidic acid (TCA) and subjected to 10–20% gradient SDS/PAGE. The Hcp polyclonal antibodies were generated by New England Peptide (Gardner, MA).

### Plaque formation assay

Two millilitre bacteria grown in LB broth for 16-hour were pelleted by centrifugation at 7600 rpm for 5 min, washed once, and resuspended in the same volume of SorC buffer. One hundred microliters of the bacteria were plated on SM/5 agar plate. *Dictyostelium* amoebae cells from mid-logarithmic cultures were collected by centrifugation, washed once with SorC buffer, and different number of amoebae cells (5×10^4^; 1×10^4^; 5×10^3^; 3×10^3^; 1×10^3^; 5×10^2^; 1×10^2^; 5×10^1^; 1×10^1^; 5×10^0^, respectively) in 5 µl SorC buffer were deposited on the top of the agar plates with *V. cholerae* wild type and different mutants. The plates were allowed to dry under a sterile flow of air and then were incubated at 22°C for 3–5 days. The plaques formed by *Dictyostelium* amoebae were recorded (see Fig. 3A for examples of plaque visualization). The least number of *Dictyostelium* amoebae cells deposited above that was able to form plaque on the bacterial lawns was defined as the minimum number of *Dictostelium* cells required for plaque formation in this study.

### Bacterial killing assay

The bacterial killing experiment was performed as described [9]. Briefly, *V. cholerae* and *E. coli* K12 CC114 were mixed at a multiplicity of infection (MOI) of 10. Twenty microliters of the mixture was spotted on LB agar plate and was incubated at 37°C for 3.5 hours. Bacterial spots were harvested and resuspended in 1 ml of LB broth. The colony-forming unit (cfu) per milliliter of surviving *V. cholerae* and *E. coli* were measured by a serial dilution and selective growth on LB agar plates containing 100 µg/ml streptomycin (for *V. cholerae*) and 12 µg/ml tetracycline (for *E.coli*) respectively.

### Actin cross-linking assay

Actin cross-linking assay was performed as described previously [5]. Briefly, J774 cells were seeded into six-well tissue culture plates at a density of 10^6^ cells per well. After 16-hour incubation at 37°C, cells were infected with various *V. cholerae* strains. The infections were performed at a MOI of 10 for 3-hour. Cells were harvested and resuspended in 50 µl of 2× loading buffer, and 10 µl each sample was analyzed by western blot using actin antiserum with the dilution at 1∶1,000.

### β-galactosidase assays

Bacteria were grown in LB broth overnight at 37°C. Beta-galactosidase activities were determined with cells permeabilized with SDS and chloroform as described by Miller [42].

## Supporting Information

Table S1 Properties of proteins encoded in *vgrG-1* and *vgrG-2* putative operons.
(DOC)

Table S2 The first ten hits of VCA0020 analyzed by HHPRED.
(DOC)

Table S3 The first ten hits of VCA0021 analyzed by HHPRED.
(DOC)

Table S4 Primers used for non-polar deletion mutants in this study.
(DOC)
