# Supplementary material for: Genetic Analysis of Anti-Amoebae and Anti-Bacterial Activities of the Type VI Secretion System in Vibrio cholerae
Source: PLoS One. 2011 Aug 31;6(8):e23876. doi: 10.1371/journal.pone.0023876 (PMC3166118; doi:10.1371/journal.pone.0023876)
Supplement: Table S1 — Properties of proteins encoded in vgrG-1 and vgrG-2 putative operons. (DOC) [file pone.0023876.s001.doc]

**Table S1.** Properties of proteins encoded in *vgrG-1* and *vgrG-2* putative operons

| Protein | Protein length  (amino acids) | Conserved domain | Function |
| --- | --- | --- | --- |
| VCA0019 | 295 | No | Uncharacterized |
| VCA0020 | 1085 | No | Uncharacterized |
| VCA0021 | 242 | No | Uncharacterized |
| VC1417 | 286 | No | Uncharacterized |
| VC1418 | 641 | COG3675; Esterase_Lipase superfamily | Uncharacterized |
| VC1419 | 247 | No | Uncharacterized |
| VC1420 | 245 | DUF2931 | Uncharacterized |
| VC1421 | 212 | LysE superfamily | Uncharacterized |
