# Supplementary material for: Genetic Analysis of Anti-Amoebae and Anti-Bacterial Activities of the Type VI Secretion System in Vibrio cholerae
Source: PLoS One. 2011 Aug 31;6(8):e23876. doi: 10.1371/journal.pone.0023876 (PMC3166118; doi:10.1371/journal.pone.0023876)
Supplement: Table S2 — The first ten hits of VCA0020 analyzed by HHPRED. (DOC) [file pone.0023876.s002.doc]

**Table** **S2.** The first ten hits of VCA0020 analyzed by HHPRED

| No. | Hit | Prob | E-Value | P-Value | Query HMM | Template HMM |
| --- | --- | --- | --- | --- | --- | --- |
| 1 | RhoGEF and PH domain containing 6, pleckstrin homology domain | 75.1 | 48 | 0.00039 | 75-177 | 9-109 |
| 2 | RhoGEF and PH domain containing protein 6 | 74.5 | 32 | 0.00026 | 74-177 | 8-109 |
| 3 | Tapp1 | 73.9 | 24 | 0.00019 | 75-169 | 4-98 |
| 4 | Pleckstrin | 67.9 | 75 | 0.00061 | 73-182 | 4-112 |
| 5 | Colicin pore forming domain | 67.5 | 2.70E+02 | 0.0022 | 773-915 | 56-182 |
| 6 | Variant surface antigen, stevor family | 66.8 | 4.90E+02 | 0.0039 | 797-919 | 185-311 |
| 7 | Src-associated adaptor protein Skap2 | 66.5 | 53 | 0.00043 | 73-167 | 8-107 |
| 8 | KIAA0640 protein; PH domain | 61.2 | 73 | 0.0006 | 75-169 | 9-101 |
| 9 | Src kinase-associated phosphoprotein SKAP55 (SCAP1) | 59.3 | 73 | 0.00059 | 75-166 | 2-98 |
| 10 | Phosphoinositide phospholipase C | 57.4 | 59 | 0.00048 | 74-114 | 1-43 |
