# Supplementary material for: Genetic Analysis of Anti-Amoebae and Anti-Bacterial Activities of the Type VI Secretion System in Vibrio cholerae
Source: PLoS One. 2011 Aug 31;6(8):e23876. doi: 10.1371/journal.pone.0023876 (PMC3166118; doi:10.1371/journal.pone.0023876)
Supplement: Table S3 — The first ten hits of VCA0021 analyzed by HHPRED. (DOC) [file pone.0023876.s003.doc]

**Table** **S3.** The first ten hits of VCA0021 analyzed by HHPRED

| No. | Hit | Prob | E-Value | P-Value | Query HMM | Template HMM |
| --- | --- | --- | --- | --- | --- | --- |
| 1 | SseC Secretion system effector C | 89.2 | 1 | 8.30E-06 | 114-147 | 58-92 |
| 2 | DUF3040 Protein of unknown function | 73.2 | 8.7 | 7.00E-05 | 3-73 | 6-76 |
| 3 | Predicted membrane protein (DUF2208) | 68.6 | 79 | 0.00063 | 13-108 | 56-182 |
| 4 | Aluminum activated malate transporter | 62.3 | 80 | 0.00065 | 38-135 | 66-170 |
| 5 | Bacteriocin AS-48 | 60.7 | 41 | 0.00033 | 103-148 | 3-44 |
| 6 | EspD | 59.4 | 23 | 0.00019 | 114-146 | 178-213 |
| 7 | Photosystem I P subunit | 56.1 | 70 | 0.00056 | 24-89 | 87-149 |
| 8 | UPF0239: Uncharacterized protein family | 56 | 32 | 0.00026 | 107-135 | 14-45 |
| 9 | DUF2208 Predicted membrane protein | 54.4 | 2.30E+02 | 0.0018 | 13-108 | 56-182 |
| 10 | Aminoglycoside/multidrug efflux system | 52.7 | 3.50E+02 | 0.0028 | 5-148 | 307-468 |
