# Supplementary material for: Genetic Analysis of Anti-Amoebae and Anti-Bacterial Activities of the Type VI Secretion System in Vibrio cholerae
Source: PLoS One. 2011 Aug 31;6(8):e23876. doi: 10.1371/journal.pone.0023876 (PMC3166118; doi:10.1371/journal.pone.0023876)
Supplement: Table S4 — Primers used for non-polar deletion mutants in this study. (DOC) [file pone.0023876.s004.doc]

**Table S4.** Primers used for non-polar deletion mutants in this study

| VCA0105-for | A**TCTAGA**TATGCTCATGATGATGAAC |
| --- | --- |
| VCA0105-int-rev | CCGTCCCAGAATTCTGTCCTCATCGGTTAGT |
| VCA0105-int-for | AGGACAGAATTCTGGGACGGTGATGATTGA |
| VCA0105-rev | A**TCTAGA**TGTTCAAATTCAACTGCA |
| VCA0106-for | A**TCTAGA**TTGCAGCGATATGGGTTACG |
| VCA0106-int-rev | CAGGGAACTAGATGAAAATCAGAGTCTTA |
| VCA0106-int-for | GATTTTCATCTAGTTCCCTGTTAAGTATT |
| VCA0106-rev | A**TCTAGA**TGCCTTGAGCAAGCTGTGGC |
| VCA0107-for | A**TCTAGA**GCGGTATTCTCAGTGGACAG |
| VCA0107-int-rev | GTGGCTCTTCTACACTTCCTTCTTTAGACA |
| VCA0107-int-for | AGGAAGTGTAGAAGAGCCACAAGCGTAAGC |
| VCA0107-rev | A**TCTAGA**GATCGTAAGGAACACGCAGC |
| VCA0108-for | A**TCTAGA**TGATAAGTTTGCATAATAAGCC |
| VCA0108-int-rev | CAAGACGTCCCCTTTCCAATACCTTTTCAGT |
| VCA0108-int-for | TATTGGAAAGGGGACGTCTTGATCAAGCCTG |
| VCA0108-rev | A**TCTAGA**ACAGCATGTTAATGATGGAGT |
| VCA0109-for | A**TCTAGA**TGAAGCTGCTGCAATACATG |
| VCA0109-in-rev | AAAACACTCGGTACGTCATAATCAGGCTTG |
| VCA0109-int-for | TATGACGTACCGAGTGTTTTAACCTCTATG |
| VCA0109-rev | A**TCTAGA**GACGATTCGATACTCTGAAC |
| VCA0110-for | A**TCTAGA**CTGATCACGGACCGTAAAGA |
| VCA0110-int-rev | CGTAAAGTGCGTCTTGCGTCATAGAGGTTA |
| VCA0110-int-for | GACGCAAGACGCACTTTACGCCAGCATCAA |
| VCA0110-rev | A**TCTAGA**GCAAAACGACTGCGAGATAA |
| VCA0111-for | A**TCTAGA**CGCGAGTGCGTCAGGACAAT |
| VCA0111-int-rev | GGGCGATATTATTCAGATCAACGGCTGCAT |
| VCA0111-int-for | TGATCTGAATAATATCGCCCTCGGCTGGAC |
| VCA0111-rev | A**TCTAGA**GGTTAGCTTGGCTGGTTGAG |
| VCA0112-for | A**TCTAGA**TACAACTTCTACCAACTG |
| VCA0112-int-rev | GCTTCTCGCGGGTTATTGCCTTACTTGAAT |
| VCA0112-int-for | GGCAATAACCCGCGAGAAGCAACTGGAGCT |
| VCA0112-rev | A**TCTAGA**TCGAGACGACGGCTGACAT |
| VCA0113-for | A**TCTAGA**CGTTATCCGCCCAACCTAG |
| VCA0113-int-rev | ATCCAATTTGGTTATAGCTCCAGTTGCT |
| VCA0113-int-for | AGCTATAACCAAATTGGATAGGGTGGAATA |
| VCA0113-rev | A**TCTAGA**GCTCAAGCTCTGACGCAAG |
| VCA0114-for | A**TCTAGA**GAGCTGGGTGAGTCTTTGCA |
| VCA0114-int-rev | ATCGCCCATGCCATCGTTTGTTATTCCACCC |
| VCA0114-int-for | ACAAACGATGGCATGGGCGATGTTGACTCAT |
| VCA0114-rev | A**TCTAGA**AGAGGCCGCTTGCTCCAGAG |
| VCA0116-for | A**TCTAGA**GTCGCACCGCGCCAATTGCCT |
| VCA0116-int-rev | GCTCAACGGGGATCACAACGTACCACCTAG |
| VCA0116-int-for | CGTTGTGATCCCCGTTGAGCGCATTCGTTTA |

**Table S4.** Primers used for non-polar deletion mutants in this study (continued)

| VCA0116-rev | A**TCTAGA**TGTGCGATAAGTCCCTGTTT |
| --- | --- |
| VCA0118-for | A**TCTAGA**GATTGTCTCTGCCGCTAACT |
| VCA0118-int-rev | CTTACCAACCTCTCATGGGGTTTTGATCTC |
| VCA0118-int-for | CCCCATGAGAGGTTGGTAAGGAGCCCGTAT |
| VCA0118-rev | A**TCTAGA**TGTAACTCATCCACTGTCCT |
| VCA0119-for | A**TCTAGA**GTTGCTTTGCCCATCGTTTA |
| VCA0119-int-rev | TTGTTTACTCTTCCATACGGGCTCCTTACC |
| VCA0119-int-for | CCGTATGGAAGAGTAAACAAGGACACAATT |
| VCA0119-rev | A**TCTAGA**CCAACACCTCTTCGCGCTGA |
| VCA0121-for | A**TCTAGA**GTCTTGGCTTCGGTGGATGA |
| VCA0121-int-rev | TCAGCTATCACGCTCTGGCAACCTATTAA |
| VCA0121-int-for | TGCCAGAGCGTGATAGCTGAAGGAAGAGG |
| VCA0121-rev | A**TCTAGA**TAGCTCGGTATGGCTCACTT |
| VCA0122-for | A**TCTAGA**TCGCTGCGAGATCTGTATCG |
| VCA0122-int-for | TTGCTCAGTGACATATCGAGTGCTATCGTC |
| VCA0122-int-rev | CTCGATATGTCACTGAGCAATCCCTTGCCC |
| VCA0122-rev | A**TCTAGA**AGGCCTTGCCATTTGCATCG |
| VCA0020-for | A**TCTAGA**GTGTGTGAAGTGTGTGAAGAG |
| VCA0020-int-rev | CGAGATTTCTACTCATCCTTGTACCTCCTGT |
| VCA0020-int-for | AAGGATGAGTAGAAATCTCGTTGTAGGAAA |
| VCA0020-rev | A**TCTAGA**GAATCACTTGTACGGCTAAA |
| VC1418-for | A**TCTAGA**ACTCCATGAATCAACCTCCC |
| VC1418-int-rev | GCTGTGAAATCATGGTTATCCCCTTAGTTC |
| VC1418-int-for | GATAACCATGATTTCACAGCAAACCTTACC |
| VC1418-rev | A**TCTAGA**CTTCCTTGATTGTATTATCA |
| VCA19-21-for | A**TCTAGA**AGTGGTGCAACGACCTACAA |
| VCA19-21-int-rev | TTCTGAGCTACATTAATTTCCCTTGGCCTC |
| VCA19-21-int-for | GAAATTAATGTAGCTCAGAACTACTTACGC |
| VCA19-21-rev | A**TCTAGA**GCGTTGTTATTGGCGATGCT |
| VC17-21-for | A**TCTAGA**TGAGCTACCCTTGTGAAATG |
| VC17-21-int-rev | CAAGCTAGGCCCACTAAGCAATAATGCGTT |
| VC17-21-int-for | TGCTTAGTGGGCCTAGCTTGTAATAAAGGT |
| VC17-21-rev | A**TCTAGA**ATACGCAGAATGCTTACTCG |
| epsD-for | A**TCTAGA**TAGTTGCTTCGGCGTGGACG |
| epsD-int-rev | GCTTGGCTTCCTCAAGATTGCGTCCCACAA |
| epsD-int-for | CAATCTTGAGGAAGCCAAGCAATGACCGAA |
| epsD-rev | A**TCTAGA**CTGACCGATACCATCAATAT |
